# ATF3 is a neuron‐specific biomarker for spinal cord injury and ischaemic stroke

**DOI:** 10.1002/ctm2.1650

**Published:** 2024-04-22

**Authors:** Jonathan Z. Pan, Zhanqiang Wang, Wei Sun, Peipei Pan, Wei Li, Yongtao Sun, Shoulin Chen, Amity Lin, Wulin Tan, Liangliang He, Jacob Greene, Virginia Yao, Lijun An, Rich Liang, Qifeng Li, Jessica Yu, Lingyi Zhang, Nikolaos Kyritsis, Xuan Duong Fernandez, Sara Moncivais, Esmeralda Mendoza, Pamela Fung, Gongming Wang, Xinhuan Niu, Qihang Du, Zhaoyang Xiao, Yuwen Chang, Peiyuan Lv, J. Russell Huie, Abel Torres‐Espin, Adam R. Ferguson, Debra D. Hemmerle, Jason F. Talbott, Philip R. Weinstein, Lisa U. Pascual, Vineeta Singh, Anthony M. DiGiorgio, Rajiv Saigal, William D. Whetstone, Geoffrey T. Manley, Sanjay S. Dhall, Jacqueline C. Bresnahan, Mervyn Maze, Xiangning Jiang, Neel S. Singhal, Michael S. Beattie, Hua Su, Zhonghui Guan

**Affiliations:** ^1^ Department of Anesthesia and Perioperative Care University of California San Francisco San Francisco California USA; ^2^ Center for Cerebrovascular Research University of California San Francisco San Francisco California USA; ^3^ Department of Neurology Cangzhou People's Hospital Cangzhou China; ^4^ Department of Anesthesiology Shandong Provincial Hospital, Shandong University Jinan China; ^5^ Department of Anesthesiology Qianfoshan Hospital, Shandong University Jinan China; ^6^ Department of Anesthesiology The Second Affiliated Hospital, Nanchang University Nanchang China; ^7^ Department of Anesthesiology Guangzhou Medical University Guangzhou China; ^8^ Department of Pain Management Xuanwu Hospital, Capital Medical University Beijing China; ^9^ Medical School University of California San Francisco San Francisco California USA; ^10^ Department of Anesthesiology No. 1 People's Hospital Huaian China; ^11^ Department of Neurosurgery Tianjin Medical University General Hospital Tianjin China; ^12^ Department of Neurological Surgery University of California San Francisco San Francisco California USA; ^13^ Brain and Spinal Injury Center University of California San Francisco San Francisco California USA; ^14^ Department of Anesthesiology The Second Affiliated Hospital, Dalian Medical University Dalian China; ^15^ Department of Neurology Hebei Medical University Shijiazhuang China; ^16^ Department of Radiology University of California San Francisco San Francisco California USA; ^17^ Department of Orthopedic Surgery Orthopaedic Trauma Institute University of California San Francisco San Francisco California USA; ^18^ Department of Neurology University of California San Francisco San Francisco California USA; ^19^ Department of Emergency Medicine University of California San Francisco San Francisco California USA; ^20^ Department of Neurosurgery Harbor UCLA Medical Center Torrance California USA

**Keywords:** activating transcription factor 3 (ATF3), biomarker, neuronal injury, neuroprotection, spinal cord injury, stroke

## Abstract

**Background:**

Although many molecules have been investigated as biomarkers for spinal cord injury (SCI) or ischemic stroke, none of them are specifically induced in central nervous system (CNS) neurons following injuries with low baseline expression. However, neuronal injury constitutes a major pathology associated with SCI or stroke and strongly correlates with neurological outcomes. Biomarkers characterized by low baseline expression and specific induction in neurons post‐injury are likely to better correlate with injury severity and recovery, demonstrating higher sensitivity and specificity for CNS injuries compared to non‐neuronal markers or pan‐neuronal markers with constitutive expressions.

**Methods:**

In animal studies, young adult wildtype and global *Atf3* knockout mice underwent unilateral cervical 5 (C5) SCI or permanent distal middle cerebral artery occlusion (pMCAO). Gene expression was assessed using RNA‐sequencing and qRT‐PCR, while protein expression was detected through immunostaining. Serum ATF3 levels in animal models and clinical human samples were measured using commercially available enzyme‐linked immune‐sorbent assay (ELISA) kits.

**Results:**

Activating transcription factor 3 (ATF3), a molecular marker for injured dorsal root ganglion sensory neurons in the peripheral nervous system, was not expressed in spinal cord or cortex of naïve mice but was induced specifically in neurons of the spinal cord or cortex within 1 day after SCI or ischemic stroke, respectively. Additionally, ATF3 protein levels in mouse blood significantly increased 1 day after SCI or ischemic stroke. Importantly, ATF3 protein levels in human serum were elevated in clinical patients within 24 hours after SCI or ischemic stroke. Moreover, Atf3 knockout mice, compared to the wildtype mice, exhibited worse neurological outcomes and larger damage regions after SCI or ischemic stroke, indicating that ATF3 has a neuroprotective function.

**Conclusions:**

ATF3 is an easily measurable, neuron‐specific biomarker for clinical SCI and ischemic stroke, with neuroprotective properties.

**Highlights:**

- ATF3 was induced specifically in neurons of the spinal cord or cortex within 1 day after SCI or ischemic stroke, respectively.
- Serum ATF3 protein levels are elevated in clinical patients within 24 hours after SCI or ischemic stroke.
- ATF3 exhibits neuroprotective properties, as evidenced by the worse neurological outcomes and larger damage regions observed in *Atf3* knockout mice compared to wildtype mice following SCI or ischemic stroke.

## Graphical abstract

• Activating transcription factor 3 (ATF3) is induced in spinal cord and cortex neurons 1 day after spinal cord injury (SCI) and ischaemic stroke, respectively.

• Serum ATF3 is elevated in patients 1 day after SCI or ischaemic stroke, and this elevation correlates linearly with the severity of SCI and ischaemic stroke.

• ATF3 is neuroprotective in SCI and ischaemic stroke.

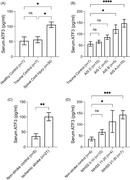

## 1 INTRODUCTION

Extensive work has identified many potential biomarkers for patients with acute central nervous system (CNS) injuries.^1^, ^2^, ^3^, ^4^, ^5^ The most promising candidates include glial fibrillary acidic protein (GFAP), neurofilament protein (NF), ubiquitin C‐terminal hydrolase L1 (UCH‐L1), S100b, neuron‐specific enolase (NSE) and RNA.^3^, ^5^, ^6^, ^7^, ^8^, ^9^ However, GFAP and S100b are expressed in astrocytes, while NF, UCH‐L1 and NSE are constitutively expressed in all neurons, regardless of whether the neurons are injured or not, and RNA biomarker candidates are expressed in white blood cells.^3^, ^5^, ^6^, ^7^, ^8^, ^9^ In other words, none of the biomarkers currently investigated have low baseline expression levels in a healthy state but are induced specifically in CNS neurons after CNS injuries. On the other hand, as neuronal injury is one of the major pathologies caused by CNS injuries, such as spinal cord injury (SCI) or stroke and strongly correlates with the neurological outcomes, biomarkers that have low baseline expression and are induced specifically in the neurons after injury are likely to correlate better with injury severity and recovery. Such biomarkers are also likely to have higher sensitivity and specificity for CNS injuries compared to non‐neuronal markers or pan‐neuronal markers with constitutive expressions.

Activating transcription factor 3 (ATF3), an early response gene, is a member of cAMP response element binding (CREB) family transcriptional factors.^10^, ^11^ ATF3 is considered as a molecular marker for stressed or injured cells^12^ because its expression level is very low in normal tissue, and it is induced by stress and pathological stimuli.^13^ For example, in the peripheral nervous system dorsal root ganglion (DRG) sensory and the spinal cord motor neurons, these is little baseline expression of ATF3, but it is rapidly induced in the injured sensory and motor neurons shortly after peripheral nerve injury.^11^, ^14^ In peripheral DRG sensory neurons, ATF3 promotes nerve regeneration because overexpression of ATF3 enhances the axonal regrowth of the injured sensory neurons.^15^, ^16^

Here, we report that ATF3 also has little expression levels in naïve CNS and is induced in CNS neurons after CNS injuries. Specifically, we demonstrate that ATF3 is induced exclusively in mouse spinal cord or cortex neurons shortly after SCI or ischaemic stroke, respectively, and its levels in the blood are elevated in mice after SCI or ischaemic stroke. Importantly, we provide evidence that human serum ATF3 is elevated in clinical patients within 24 h after SCI or ischaemic stroke. Mechanistically, we reveal that ATF3 exhibits a neuroprotective function after SCI or ischaemic stroke in mouse models. Thus, ATF3 is likely a clinical neuron‐specific biomarker that can be used to assess the extent of neuronal injury in patients with acute SCI and ischaemic stroke.

## 2 MATERIALS AND METHODS

### 2.1 Animals

The animal studies were approved by the local institutional animal care and use program. C57BL/6J wild‐type (WT) mice and Long‐Evans rats were purchased from the Jackson Laboratory and Charles River, respectively. The immunohistochemistry study of SCI was conducted in rat, and all other animal studies were conducted in mouse. *Atf3* knockout (KO) mice with a C57/BL/6J background were provided by Dr. Tsonwin Hai.^17^ The animals were housed with ad libitum access to food and water. Eight to 10‐week‐old WT and *Atf3* KO mice were randomly assigned to different experimental groups. For the stroke model, both male and female animals were used and analysed together. For the SCI model, only female animals were included due to fewer post‐injury complications, especially urinary tract infection.^18^, ^19^

### 2.2 Neurological models

SCI was performed as previously described.^20^ All surgical procedures were carried out aseptically. Animals received cefazolin at a dosage of 50 mg/kg prior to procedure and again 1 day after the SCI procedure. Animals were anesthetised with 2% isoflurane, and buprenorphine (.1 mg/kg) was administered before surgery and the next day after surgery. A dorsal midline skin incision was made, followed by dissection of connective tissue and muscle layers, and then a C5 laminectomy was performed. Unilateral (right) cervical contusion injury at 30 Kdyne (mouse) or 75 Kdyne (rat) was induced using an Infinite Horizon impactor device with an appropriate impactor tip diameter of 1 mm (mouse) or 2 mm (rat). The control group underwent all procedures with no contusion. Following the procedure, muscle layers were closed with sutures and the skin with clips. For the first day post‐SCI, animals were placed in an incubator at 37°C and monitored closely. Subsequently, animals were checked twice daily for wound healing and bladder function for up to 7 days following the SCI procedure.

Permanent distal middle cerebral artery occlusion (pMCAO) was performed as previously described.^21^ In brief, a 1.0 cm incision was made from the left orbit to the left ear, followed by a craniotomy (2 mm^2^) to expose distal branches of the middle cerebral artery (MCA). The MCA was then electrically coagulated proximal to the pyriform branch. Cerebral blood flow on the brain's surface was monitored by Doppler flow‐meter (Vasamedics). Mice were excluded if the reduction in ischaemic core blood flow was <15% of baseline or if extensive bleeding occurred. Mice recovered from anesthesia in warm cages. All surgeries were performed aseptically under 2% isoflurane. Buprenorphine (.1 mg/kg) was administered before and 6 h after the surgery, and as needed thereafter. Temperature was maintained at 37°C using a thermal blanket during surgery. Sham control mice underwent craniotomy with no arterial coagulation and received the same treatments as mice subjected to pMCAO.

### 2.3 Behavioural tests

We employed a paw placement in a cylinder task^20^ on WT and *Atf3* KO mice after SCI. The frequency of contralateral (left) forepaw placement is calculated as a proportion of total placements and is used as an indicator of recovery on the ipsilateral (right) side. In normal animals, both left and right paws are placed approximately equally; after injury, the paw contralateral to the SCI is preferentially used. The test was performed before SCI (baseline) and 2, 7 and 14 days post‐SCI.

We also employed two behavioural tests on pMCAO mice: (1) *adhesive removal test* was performed to assess potential somatosensory dysfunction.^22^ In brief, a .3 × .3 cm adhesive tape was placed on the forepaws, and the time taken to remove the tape was recorded, with a cut‐off time of 120 s. Mice underwent training twice daily for 4 days before the pMCAO procedure to achieve optimal performance. Adhesive removal times were recorded 1 day before pMCAO (baseline) and 3 days after pMCAO. In general, stroke mice take longer to remove tape from the paw on the side contralateral to the stroke lesion. Since the infarct in this study was on the left side, the adhesive removal time from the right paw was more relevant. (2) *Corner test* was performed as previously reported.^23^ Mice were placed between two boards measuring 30 × 20 cm. Both sides of the vibrissae were stimulated as they approached the corner. Mice then moved upward and turned to face the open end. The frequency of right and left turns would be equal in normal mice. In stroke mice, they made more left turns (ipsilateral of stroke lesion). Three sets of 10 trials were conducted, with the exclusion of turns not incorporated into rearing movements.

### 2.4 RNA preparation and quantitative reverse transcriptase polymerase chain reaction

Total RNA was prepared as previously described.^24^ RNA samples were purified from a 5 mm segment of mouse spinal cord around the contusion site (or around C5 for uninjured tissue) using Trizol (Invitrogen), RNeasy (Qiagen) and NanoDrop Lite (Thermo Scientific) quantification. We assessed mRNA expression of target genes in uninjured and injured spinal cord tissue (4 h or 1 day; *n* = 3−4/group per timepoint) using quantitative reverse transcriptase polymerase chain reaction (qRT‐PCR)^24^ with gene‐specific primer pairs. cDNA was prepared from 2 µg RNA through SuperScript II and random primers (Invitrogen). PCR was performed using 10 ng of cDNA, 50 nm of each primer and SYBR Green master mix (Applied Biosystems). qRT‐PCR products were measured using an Agilent Mx3005P Real‐Time PCR system.

### 2.5 RNA‐sequencing approach

RNA was extracted from WT sham and injured mice, quantitated, and 50 ng of RNA from each sample was submitted to the UCSF Functional Genomic Core facility for RNA‐sequencing (RNA‐Seq). We used the Illumina HiSeq 4000 sequencer (Illumina) and ran a total of 12 samples in one lane, performing 50 bp single‐ended sequencing at 30 million reads per sample. The UCSF Functional Genomic Core facility provided the data upon library submission. Subsequently, we trimmed and aligned the data against the mouse genome. Differential expression analyses, including multiple comparisons with Benjamini‒Hochberg false discovery rate^25^ (BHFDR adjusted *p* < .05), were conducted on our samples, resulting in the identification of 177 genes (160 upregulated and 17 downregulated) with more than 1.5‐fold changes (BHFDR adjusted *p* < .05). To gain further insights, these differentially expressed genes (DEG) were submitted to Metascape (metascape.org) for Gene Ontology (GO) analysis.^26^ Additionally, we generated a heatmap and a volcano plot to visualise the data using GraphPad Prism 8. We have deposited our sequence data in the Gene Expression Omnibus database, providing a valuable new resource for the SCI community (see data and materials availability).

### 2.6 Tissue processing and immunohistochemical staining

#### 2.6.1 SCI

Animals were euthanised under deep anesthesia (ketamine and xylazine) by transcardiac perfusion with .9% saline and 4% paraformaldehyde.^24^ Segments of the spinal cord were subsequently removed, post‐fixed overnight in 4% paraformaldehyde and cryoprotected in 30% sucrose for 2 days. After embedment in optimal cutting temperature (OCT) compound, the samples were horizontally sectioned into 20 µm slices, with adjacent sections divided across six sets of slides. Immunohistochemistry^24^ was conducted using a high‐throughput staining station (Sequenza; Thermo Scientific). Sections were first incubated at room temperature for 1 h with 10% normal donkey serum and .3% Triton X‐100, and then overnight with rabbit polyclonal ATF3 antibody (1:300, Novus Biologicals, Cat# NBP1‐85816), mouse monoclonal antibody for NeuN (1:500, Millipore, Cat# MAB377), mouse anti‐rat CD68 (1:500, Bio‐Rad, Cat# MCA341GA) or mouse anti‐rat GFAP (1:500, Millipore, Cat# MAB360). Following four washes with phosphate‐buffered saline (PBS), all slides were incubated for 1 h at room temperature with fluorescent secondary antibodies such as Donkey anti‐mouse 1:500 Alexa 488 and Donkey anti‐rabbit 1:500 Alexa 594‐conjugated IgG (Invitrogen, Cat# A21202 and A21207). Slides were rinsed with PBS and mounted with Vectashield containing 4',6‐diamidino‐2‐phenylindole (DAPI; Vector Laboratories). The sections were photographed using a BIOREVO all‐in‐one fluorescence microscope (BZ‐9000 Generation II, Keyence microscope) with either a ×10, ×20 or ×40 objective. The images from cross‐sections were stitched together and measured using the BZ‐9000 Generation II analyser (Keyence microscope).

#### 2.6.1 pMCAO

Mice were anesthetised and brain tissues were collected and rapidly frozen on dry ice. Brain tissue preservation was achieved without cryoprotection, which has been shown to effectively maintain tissue integrity for analysing different cells.^27^, ^28^, ^29^ Coronal sections, each 20 µm thick, were prepared between bregma −1.22 and −2.18 mm using a Leica Cryostat (CM1950). After air drying and washing with PBS, the sections were stained with primary antibodies specific to mouse CD68 rat monoclonal antibody (1:50, Bio‐Rad [Formerly AbD Serotec], MCA1957), mouse NeuN mouse monoclonal antibody (for neuronal nuclei, 1:500, Millipore, #MAB377) and mouse ATF3 rabbit monoclonal antibody (1:200, Cell Signalling, #33593) at 4°C overnight. Subsequently, they were incubated with secondary antibodies, either Alexa Fluor 594‐conjugated IgG or Alexa Fluor 488‐conjugated IgG (1:500, Invitrogen) at room temperature for 2 h. Fluoro‐Jade C (FJC, Millipore) staining was performed following the manufacturer's instruction. Sections were mounted using Vectashield containing DAPI. Although the anti‐NeuN and anti‐CD68 antibodies are raised in mouse, the staining was highly specific and no additional blocking step was required, as previously published.^27^, ^28^

### 2.7 Injury size estimation

The lesion volume of spinal cord injured mice was assessed using paraformaldehyde‐fixed tissue collected at the end of 2 weeks following mouse SCI.^24^ Eriochrome cyanine (EC) staining was employed to distinguish spinal tissues.^30^ Camera lucida drawings were created for the sections with the largest lesions, outlining intact grey and white matters as well as the lesions. Areas where normal spinal cord architectures were absent, and where demyelination or fibrosis was presented, were manually outlined from digitised images and defined as the lesion. The proportion of the ipsilateral lesion area to the contralateral normal spinal cord section was then calculated accordingly.

The infarct volumes were measured in pMCAO mice 3 days after the injury. Every 10 brain sections (spaced 200 µm apart) were chosen, stained with cresyl violet and then imaged. The infarct areas were outlined and quantified using ImageJ (National Institutes of Health [NIH]). To calculate the infarct volume, the sum of the infarct areas from all the cresyl violet‐stained sections was multiplied by 200.

### 2.8 Patients and control enrollment

The clinical procedures for the SCI and stroke studies were conducted with the approval of the Human Subjects Review Boards at the University of California San Francisco (IRB numbers: 15‐16115 and 19‐27658). Informed consent was obtained from all subjects. Our male and female SCI patients, trauma controls and healthy cohort were part of the Brain and Spinal Injury Center Transforming Research and Clinical Knowledge project^3^, ^31^ (https://clinicaltrials.gov/ct2/show/NCT04565366). Blood samples were collected from 30 SCI patients, whose American Spinal Injury Association Impairment Scale (AIS) scores ranged from D to A, at 24 h after injury (Table S1), as well as from seven trauma control patients without SCI or traumatic brain injury (TBI) at 24 h after injury and seven healthy controls. Patients presented to the emergency department with the diagnosis of traumatic SCI were enrolled with ISNCSCI examinations, blood draws and follow‐up assessments. The trauma control patients were recruited from the emergency department with traumatic but non‐CNS injuries. Similarly, both male and female ischaemic stroke patients were enrolled and blood samples were collected in the emergency room or intensive care units within 24 h of stroke (Table S2). We also collected biospecimens and demographic information for a control cohort without stroke for comparison.

### 2.9 Plasma (mouse) and serum (human) sample collection

Sham and injured mice were anesthetised and blood was collected via intracardiac puncture. Typically, 500−700 µL blood could be obtained. Blood samples were stored in plastic blood collection tubes with ethylenediaminetetraacetic acid (EDTA) (BD Vacutainer Labware Medical) on ice until centrifugation. The samples were then centrifuged at 845 *g* for 15 min, and the upper layer containing plasma was collected and stored at −80°C. Human serum samples were prepared by separating the clot using serum separator tubes. Externally threaded cryovials were used for serum storage, with serum divided into multiple 500 µL aliquots.

### 2.10 ATF3 enzyme‐linked immune‐sorbent assay

Commercial mouse or human ATF3 enzyme‐linked immune‐sorbent assay (ELISA) kits (Aviva Systems Biology, Cat# OKDD00746 and OKDD01469) were used to quantitate ATF3 protein levels in plasma (mouse samples) or serum (human samples) using standard sandwich ELISA technology.^32^ Standards and test samples were assayed following the manufacturer's procedures. Typically, all wells in the microplate were precoated with ATF3 antibody. Samples for the standard curve were reconstituted from the standard stock solution to concentrations of 1000, 500, 250, 125, 62.5, 31.2 and 15.6 pg/mL and blank (0 pg/mL). Plasma (mouse) or serum (human) samples were used without dilution, with duplicates for each standard and testing sample. The microplate was covered with a plate sealer before each incubation at 37°C. After adding the standard and testing samples into the precoated wells, the plate was incubated at 37°C for 90 min. Then, 100 µL of biotinylated detection antibody was added to each well after removing the liquid, and the samples were incubated at 37°C for another 45 min. After the aspiration/wash process was repeated for a total of three times using washing buffer, 100 µL of Avidin‐Horseradish peroxidase (HRP) conjugate was added, and the plate was incubated at 37°C for 45 min. Following another aspiration/wash process, repeated for five times, 90 µL of substrate solution was added, protected from light and the plate was incubated at 37°C for 15−25 min. Finally, 50 µL of stop solution was added and samples were measured at 450 nm using a microplate reader.

### 2.11 Statistical analyses

Two‐way analysis of variance (ANOVA) with repeated measures was used for behavioural analyses. Terminal histology, ELISA or qRT‐PCR results in both animal models and human study were analysed by either two‐way ANOVA or one‐way ANOVA for more than two experimental groups, or unpaired two‐tailed *t*‐tests for two group analyses. Normality of residuals was examined using the Shapiro–Wilk test, which revealed no significant departure from normality. GraphPad Prism 9 (GraphPad software) was used for creating graphs and performing data analyses. Data are presented as means ± SEM. Sample size was estimated based on our previous published effect sizes of injury area and functional tests in similar animal models.^24^, ^33^, ^34^ Statistically significance was defined as a *p*‐value of ≤.05.

## 3 RESULTS

### 3.1 *Atf3* gene upregulation in spinal cord tissue shortly after SCI

**FIGURE 1:**
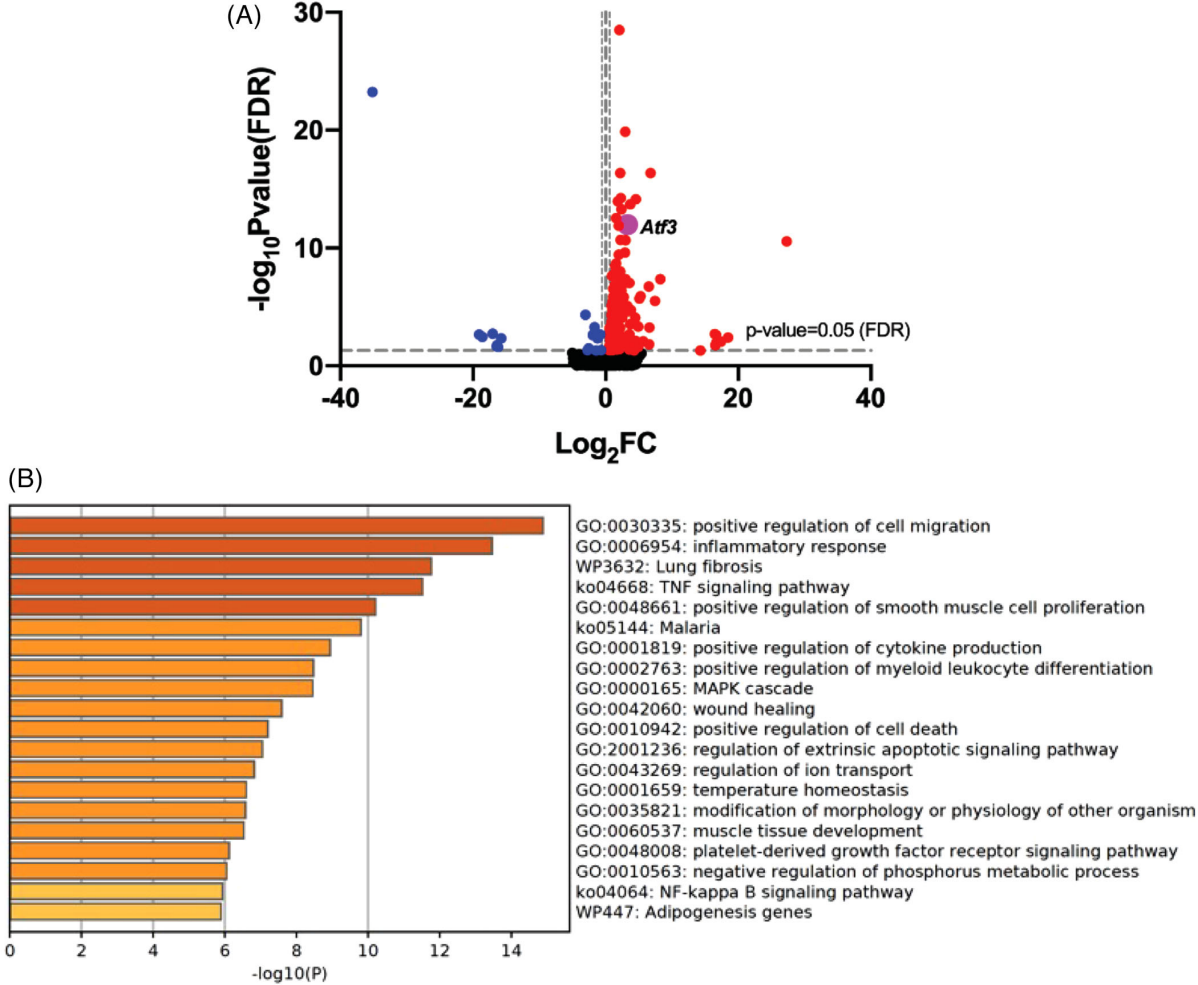
*RNA‐Seq* of mouse spinal cord after spinal cord injury. (a) Volcano plots of mouse spinal cord RNA‐sequencing (RNA‐Seq) results showing that *Atf3*, highlighted in purple, is one of the most significantly upregulated genes 4 h after spinal cord injury (SCI) (adjusted Benjamini‒Hochberg false discovery rate [BHFDR] *p* < .05). (b) Top 20 in Gene Ontology (GO) analysis of differentially expressed genes (DEG) from RNA‐Seq, showing multiple major pathways including mitogen‐activated protein kinase (MAPK) cascade, positive regulation of cell death, and regulation of extrinsic apoptotic signalling pathway, and negative regulation of phosphorus metabolic pathway.

To identify the potential biomarkers for SCI, we performed RNA‐Seq on mouse spinal cord tissues before and shortly after unilateral cervical spinal cord contusion. Our goal was to discover biomarkers in the early phase of SCI, as these early timepoints are crucial for understanding the development of pathophysiological changes in SCI when many injured neurons may still be viable and rescuable. In contrast to the previous RNA‐Seq studies of SCI, which examined the gene expression profiles in injured spinal tissue 1−28 days after SCI,^35^, ^36^, ^37^ we analysed gene expression profiles just 4 h after SCI (Figure 1a). Compared to the sham control, the injured spinal cord exhibited significant changes in 177 genes with more than 1.5‐fold changes (either up‐ or downregulated) after multiple comparison tests (BHFDR^25^ *p* < .05) (Figure S1). Among these DEG, 160 genes were upregulated and 17 were downregulated. GO analysis identified major pathways related to inflammatory responses, cellular apoptotic responses and signalling transduction pathways, all of which reflect the acute reactions to trauma in spinal tissue (Figure 1b). We found that the *Atf3* gene was one of the most upregulated genes, showing a 9.9‐fold increase as detected through RNA‐Seq (Figure 1a). This is particularly interesting because the *Atf3* gene is typically induced only in injured DRG sensory neurons and spinal motor neurons, following peripheral nerve injury.^14^

### 3.2 ATF3 protein is induced specifically in spinal cord neurons 1 day after SCI

**FIGURE 2:**
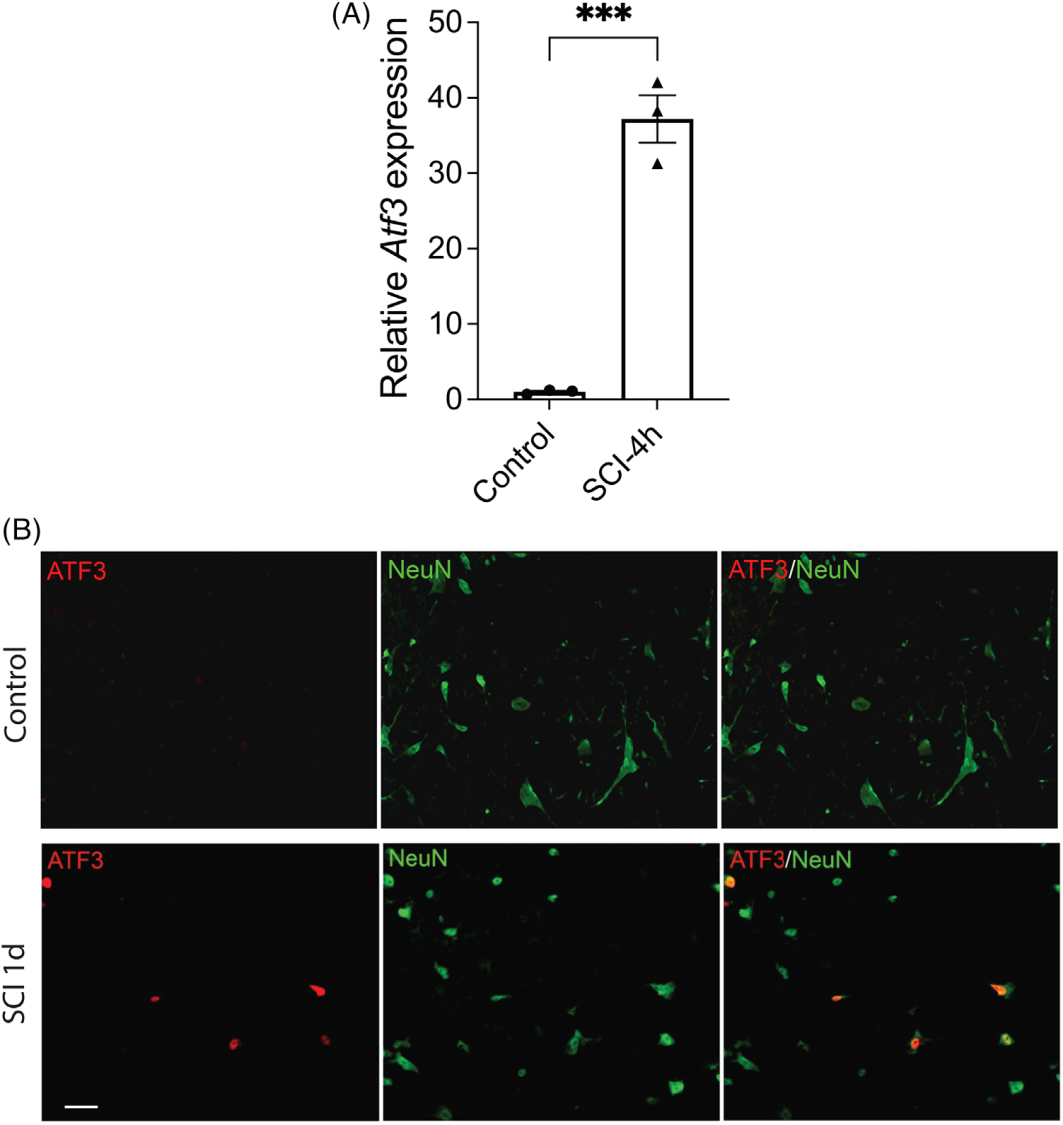
Activating transcription factor 3 (ATF3) induction in the neurons of injured hemi‐cord after spinal cord injury (SCI). (a) Quantitative reverse transcriptase polymerase chain reaction (qRT‐PCR) confirms the remarkably increased *Atf3* gene expression in mouse spinal cord 4 h after SCI. The results are normalized to *Atf3* expression in control animals. Data are presented as mean ± SEM and were analysed with unpaired two‐tailed *t*‐test, ^***^ *p* < .001, *n* = 3 in each group. (b) Representative immunohistochemical staining of ATF3 and NeuN in control and injured hemi‐cord 1 day after SCI. All ATF3^+^ cells are NeuN^+^ 1 day after SCI. Scale bar = 40 µm. The images are the magnification of the squared areas in Figure S1.

Our qRT‐PCR results confirmed that *Atf3* gene expression in the spinal cord was very low in sham controls but exhibited a dramatic upregulation (by 37‐fold) just 4 h after SCI (Figure 2a), consistent with the previous reports that *Atf3* gene is induced in spinal cord after SCI.^38^, ^39^ Because ATF3 is typically induced in sensory neurons only after peripheral nerve injury, and it is considered as a cellular marker of injured DRG sensory neurons,^14^ we were curious whether ATF3 is also induced in spinal cord neurons after SCI. Immunohistochemical (IHC) staining revealed that ATF3 protein was barely detectable in the spinal cord of control naïve uninjured animals (Figure 2b). However, it was strongly induced in NeuN^+^ neurons in the spinal cord 1 day after SCI (Figures 2b and S2) in the area surrounding the centre of the damage (Figure S3), with no detectable ATF3 signals in CD68^+^ microglia/macrophages (Figure S4a) or GFAP^+^ astrocytes (Figure S4b) 1 day after SCI. This result is consistent with the previous reports that ATF3 protein is induced in spinal cord neurons 1 day after SCI.^13^

### 3.3 ATF3 protein is induced specifically in cortex neurons 1 day after ischaemic stroke

**FIGURE 3:**
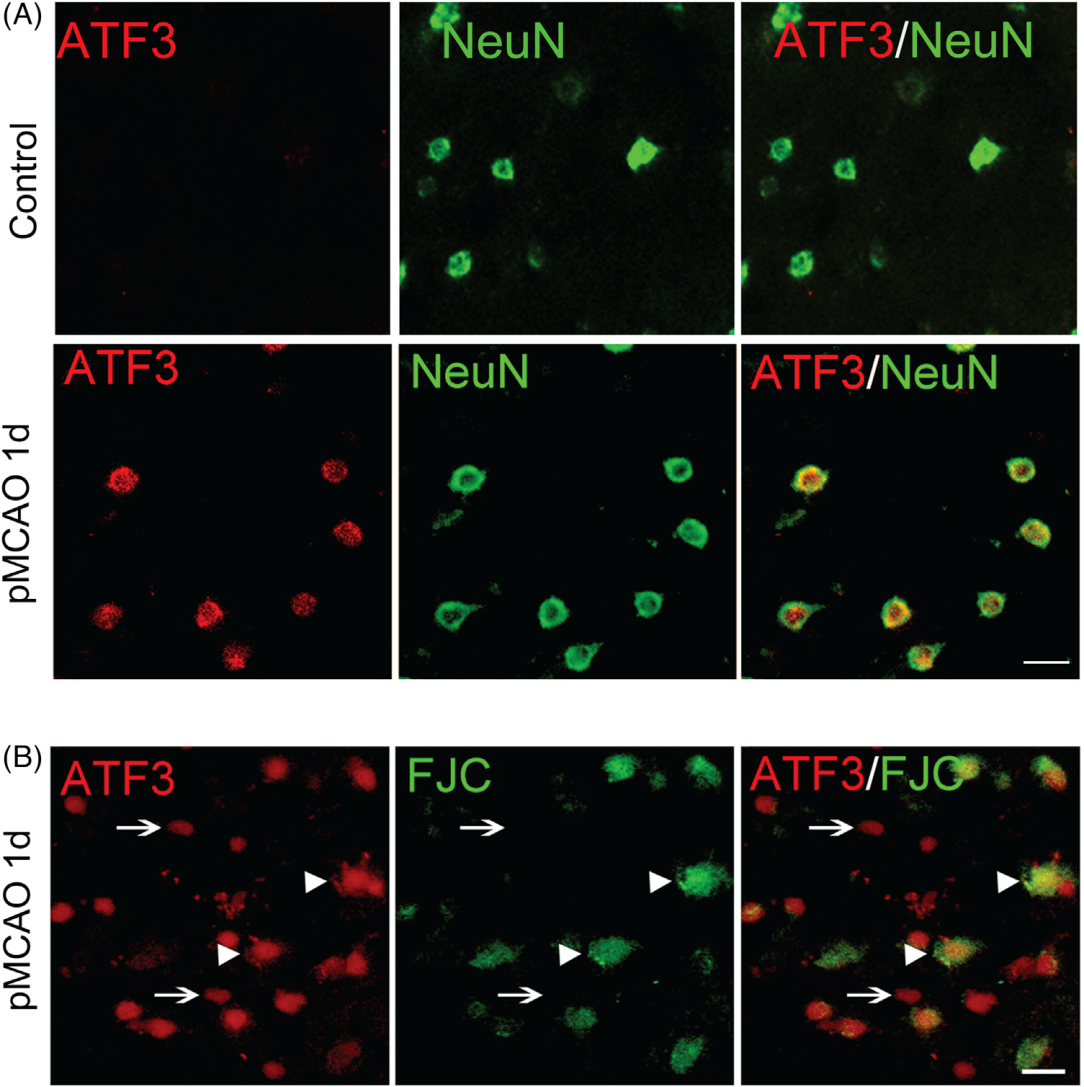
Activating transcription factor 3 (ATF3) is induced in the neurons in peri‐infarct area after ischaemic stroke. (a) Representative immunohistochemical staining of NeuN and ATF3 in control and peri‐infarct ischaemia region 1 day after permanent distal middle cerebral artery occlusion (pMCAO) in mice. All ATF3^+^ cells are NeuN^+^. (b) Representative immunohistochemical staining of ATF3 and Fluoro‐Jade C (FJC, a known marker for degenerating neurons) in the peri‐infarct ischemia region 1 day after pMCAO in mice. All FJC^+^ cells are ATF3^+^ (arrowheads), but some ATF3^+^ cells are FJC^−^ (arrows). Scale bar = 50 µm.

To investigate whether ATF3 is also induced in other neurological conditions, we expanded our study to include mice with pMCAO, an ischaemic stroke model. In fact, it has been reported that *Atf3* gene is induced after pMCAO.^40^, ^41^ Consistent with our findings in the injured spinal cord, we observed that ATF3 protein was barely detectable in the control cortex without stroke (Figure 3a). However, 1 day after pMCAO stroke, ATF3 was strongly expressed in NeuN^+^ neurons in the peri‐infarct ischaemic brain tissue, with all ATF3^+^ cells co‐expressing NeuN (Figures 3b and S5a). This result is consistent with the previous reports that ATF3 protein is induced in cortical neurons after pMCAO.^42^

### 3.4 All the degenerating neurons in cortex after ischaemic stroke are ATF3^+^

To investigate the relationship between neuronal ATF3 expression and neuronal injury, we co‐immunostained peri‐infarct ischaemic brain tissue 1 day after pMCAO with ATF3 and FJC, a known marker for degenerated neurons.^43^ We observed that all FJC^+^ cells co‐expressed ATF3, although some ATF3^+^ cells were FJC^−^ (Figures 3b and S5b). Unfortunately, Fluoro‐Jade staining is non‐specific in injured spinal cord tissues after SCI and cannot be used to label the degenerating spinal cord neurons after SCI.^44^

### 3.5 ATF3 levels in blood are elevated in mice with SCI or pMCAO ischaemic stroke

**FIGURE 4:**
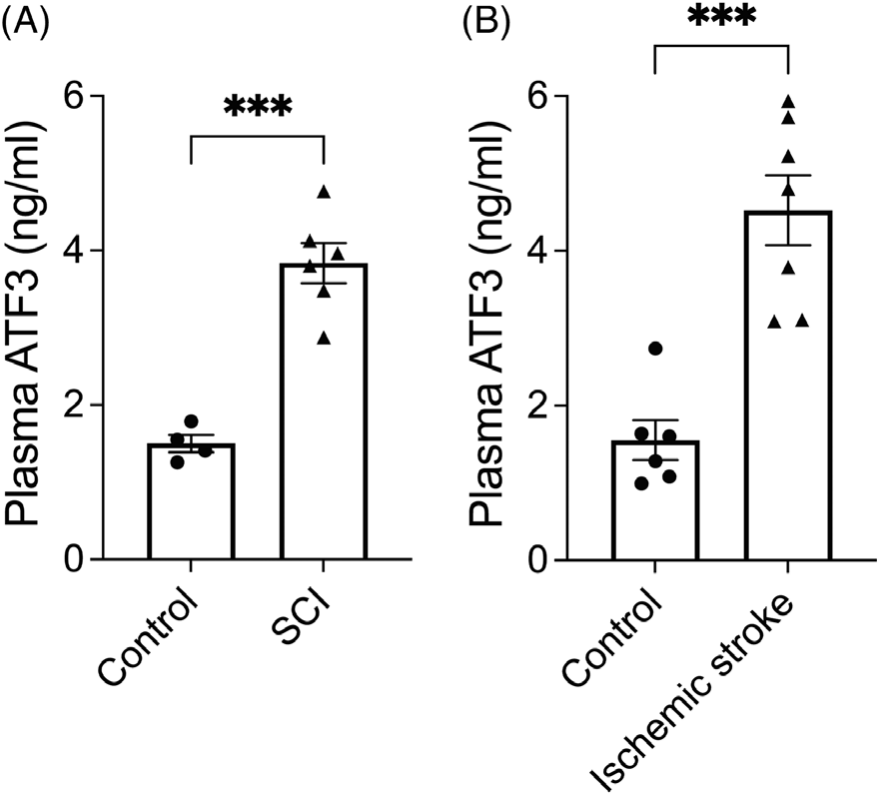
Increased plasma activating transcription factor 3 (ATF3) protein levels after rodent spinal cord injury (SCI) or ischaemic stroke. Enzyme‐linked immune‐sorbent assay (ELISA) results showing ATF3 protein level was detectable in mouse plasma, and its level was increased significantly post‐SCI (a) or ischaemic stroke (b). Data are presented as mean ± SEM and are analysed with unpaired two‐tailed *t*‐test, ^***^ *p* < .001, *n* = 4−7 in each group.

Since ATF3 was induced exclusively in neurons 1 day after SCI or ischaemic stroke, we explored the possibility of detecting ATF3 protein levels in the peripheral blood as a potential biomarker. We collected blood samples from mice 1 day after sham surgery or SCI and analysed them using a mouse ATF3 ELISA kit. Compared to sham control, mice subjected to SCI had higher levels of ATF3 protein in blood 1 day after the injury (Figure 4a). Similarly, in the mouse pMCAO model, we observed an increase in ATF3 protein level in the blood 1 day after artery occlusion (Figure 4b).

### 3.6 Serum ATF3 is elevated in clinical patients within 24 h after SCI

**FIGURE 5:**
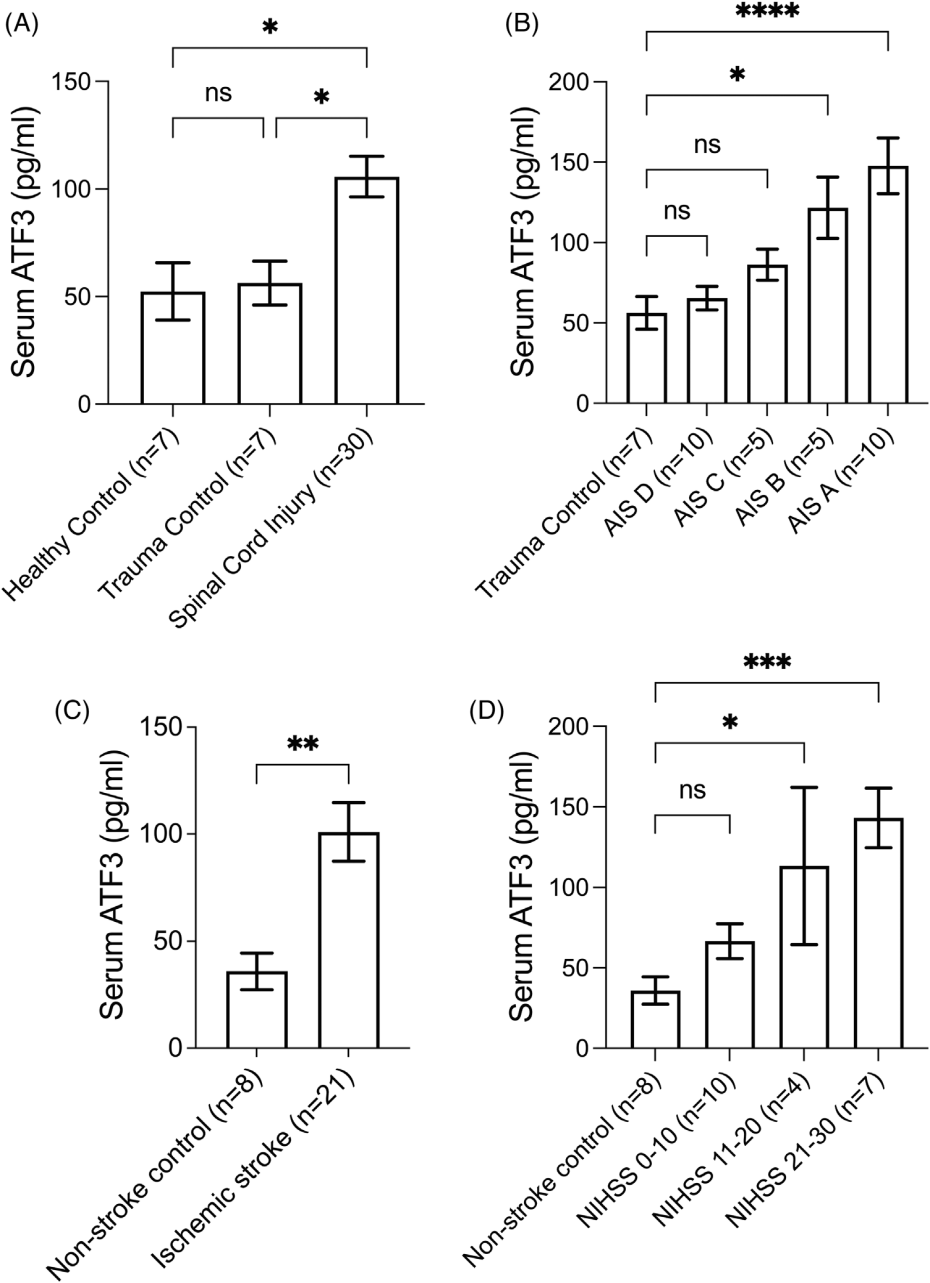
Serum activating transcription factor 3 (ATF3) is elevated in clinical spinal cord injury (SCI) and ischaemic stroke patients 24 h after injury. (a) Human serum ATF3 levels were measured using a commercially available enzyme‐linked immune‐sorbent assay (ELISA) kit in healthy control (*n* = 7), trauma control patients without SCI or traumatic brain injury (TBI) 24 h after injury (*n* = 7), and SCI patients 24 h after injury (*n* = 30). Serum ATF3 levels in SCI patients were significantly higher than those in healthy control and trauma control patients, with no statistical difference between healthy control and trauma control groups. (b) The serum ATF3 levels in patients with different severity of SCI. American Spinal Injury Association Impairment Scale (AIS) D represents mild SCI, while AIS A indicates the most severe SCI. (c) Human serum ATF3 levels were measured by ELISA from non‐stroke patient controls (*n* = 8) and patients within 24 h of ischaemic stroke (*n* = 21). Serum ATF3 levels were significantly elevated in stroke patients. (d) The serum ATF3 levels in patients with different NIH Stroke Score/Scale (NIHSS) at 24 h. Data are presented as mean ± SEM and are analysed with one‐way analysis of variance (ANOVA) with Bonferroni's multiple comparison tests (a, b and d) or unpaired two‐tailed *t*‐test (c), ^****^ *p* < .0001, ^***^ *p* < .001, ^**^ *p* < .01, ^*^ *p* < .05 and ‘ns’ as not statistically significant.

Our preclinical findings, which showed that ATF3 is elevated in mouse blood after SCI and ischaemic stroke, prompted us to investigate if serum ATF3 could serve as a biomarker for clinical patients with SCI or ischaemic stroke. Using a commercially available human ATF3 ELISA kit, we successfully measured ATF3 levels in human serum samples from healthy control people (*n* = 7), patients who were 24 h after peripheral trauma without SCI or TBI (*n* = 7) and patients who were 24 h after SCI (*n* = 30). We found that serum ATF3 levels were significantly higher in SCI patients (105.7 ± 9.5 pg/mL) compared to healthy controls (52.4 ± 13.3 pg/mL) and trauma controls (56.3 ± 10.2 pg/mL) (*p* < .05) (Figure 5a), especially in patients with moderate to severe SCI (Figure 5b). However, there was no difference between healthy controls and non‐SCI/TBI trauma control patients (*p* = .99) (Figure 5a). Among patients with AIS A (the most severe) SCI, no statistically significant differences were found in serum ATF3 levels based on sexes, ages, races, injury levels or injury categories (Figure S6).

### 3.7 Serum ATF3 is elevated in clinical patients within 24 h after ischaemic stroke

Similarly, we measured serum ATF3 levels in non‐stroke control (*n* = 8) and patients who were 24 h after ischaemic stroke (*n* = 21) and found that the serum ATF3 level was also significantly elevated in ischaemic stroke patients (101.0 ± 13.7 pg/mL) compared to non‐stroke controls (35.9 ± 8.5 pg/mL) (*p* < .01) (Figure 5c), especially in patients with moderate to high NIH Stroke Scale/Score at 24 h (Figure 5d). Therefore, our data demonstrate that serum ATF3 protein levels can be easily measured as a novel neuron‐specific biomarker for clinical patients with moderate to severe SCI or ischaemic stroke.

### 3.8 *Atf3* KO mice have impaired neurological function after SCI or ischaemic stroke

**FIGURE 6:**
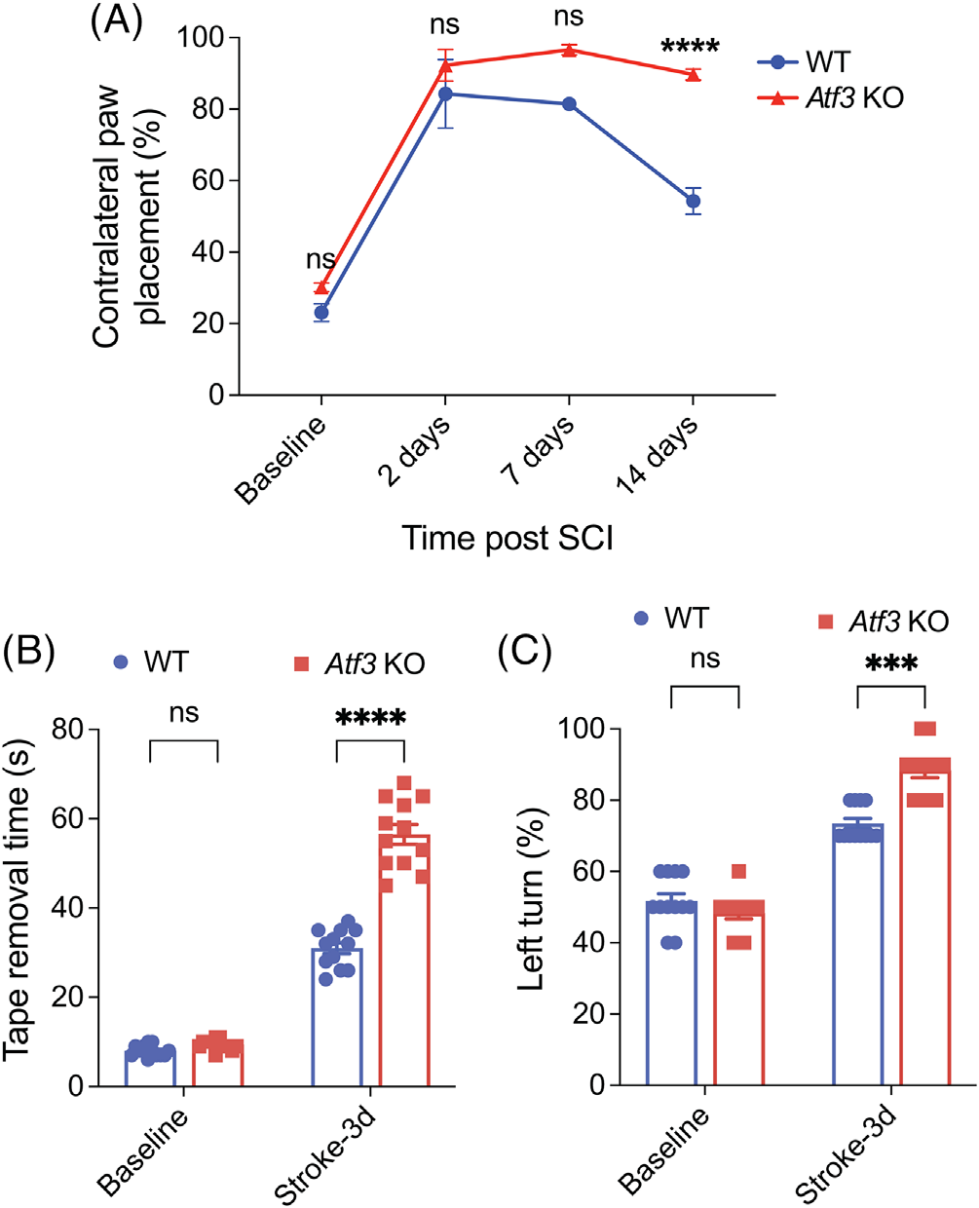
*Atf3* knockout (KO) mice had worse neurological outcomes in spinal cord injury (SCI) or ischaemic stroke models. (a) Paw placement in a cylinder task showing that *Atf3* KO mice had worse functional recovery after SCI compared to wild‐type (WT) mice. Sticker removal time from right paw (b) and quantification of left turns in corner test (c) showing that *Atf3* KO mice had more severe sensorimotor dysfunction than WT mice 3 days after left permanent distal middle cerebral artery occlusion (pMCAO). Data are presented as mean ± SEM, *n* = 7−12 in each group and are analysed with two‐way analysis of variance (ANOVA) and Sidak's multiple comparisons tests, ^****^ *p* < .0001, ^***^ *p* < .001 and ‘ns’ as not statistically significant.

To investigate the role of ATF3 in SCI, we assessed and compared functional recovery in WT and *Atf3* KO mice^17^ after SCI. Following unilateral spinal cord contusion at C5, the paw placement test^20^ revealed that *Atf3* KO mice exhibited significantly less ipsilateral weight support (increased contralateral uninjured forepaw placement against cylinder for weight support) compared to WT animals 2 weeks after injury (Figure 6a), indicating that *Atf3* KO mice had worse motor dysfunction after SCI than WT mice.

For the mice with ischaemic stroke, we conducted adhesive removal^22^ and corner tests^23^ in both WT and *Atf3* KO mice before and 3 days after left pMCAO to assess sensorimotor function. In the adhesive removal test, we observed that both WT and the *Atf3* KO mice took a similar amount of time to remove a sticker from their right paws at baseline (Figure 6b). However, 3 days after left pMCAO, *Atf3* KO mice required significantly more time than WT mice to remove stickers from their right paws (Figure 6b), indicating that *Atf3* KO mice experienced more severe sensorimotor dysfunction after ischaemic stroke than WT mice. Similarly, in the corner test, both WT and *Atf3* KO mice made approximately 50% left turns at baseline before stroke (Figure 6c), but after 3 days of left pMCAO, *Atf3* KO mice made significantly more left turns than WT mice (Figure 6c). This finding further demonstrates that knocking out *Atf3* worsens sensorimotor dysfunction after ischaemic stroke in mice.

### 3.9 *Atf3* KO mice exhibit more severe tissue damages after SCI or ischaemic stroke

**FIGURE 7:**
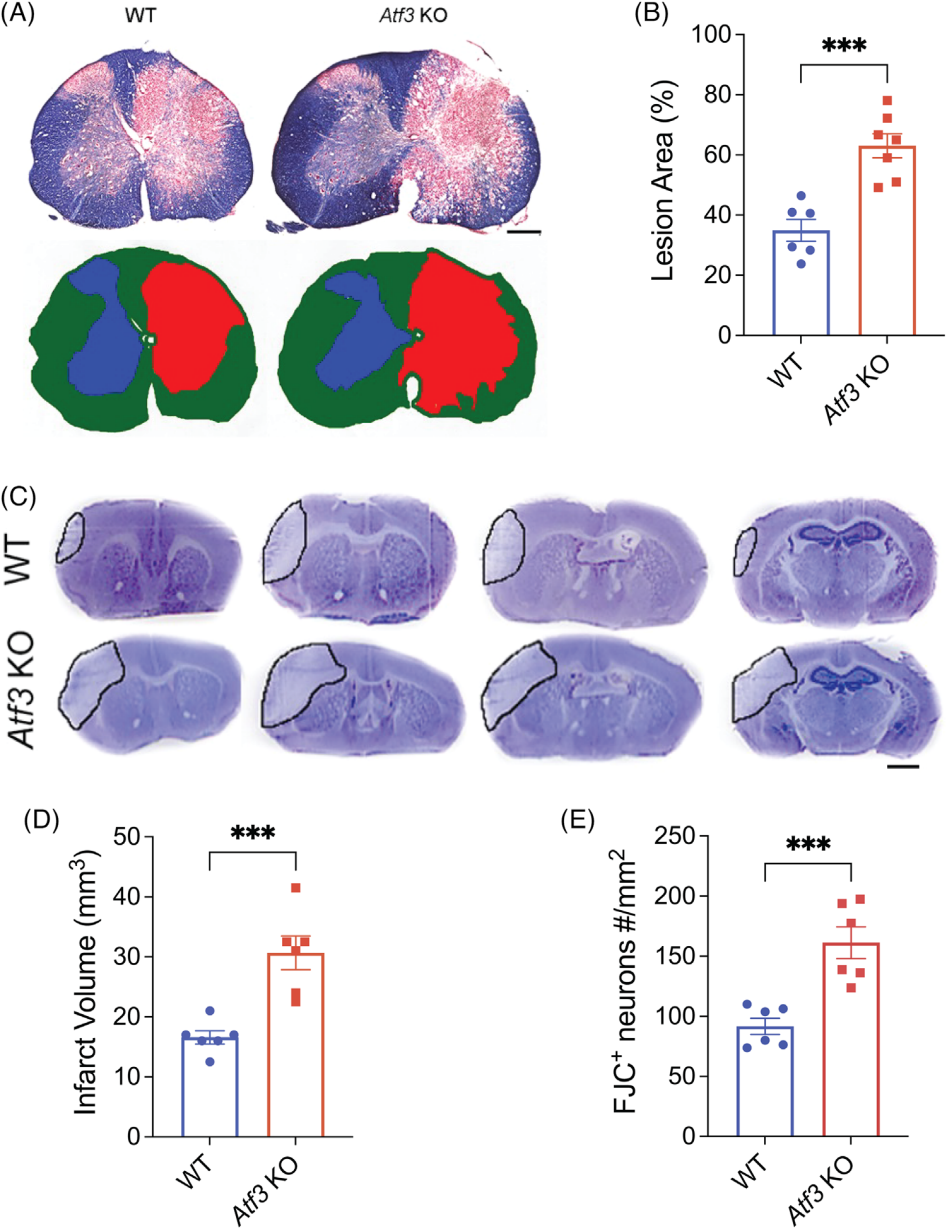
*Atf3* knockout (KO) mice had worse tissue injury after spinal cord injury (SCI) or ischaemic stroke. (a) SCI lesion area measured by Eriochrome cyanine (EC) staining, with the schematic outline, and (b) the quantification of the injury area in spinal cord of wild‐type (WT) and *Atf3* KO mice 2 weeks after SCI. Scale bar = 200 µm. The injury size, presented as the percentage of ipsilateral lesion area in total contralateral uninjured area, was larger in *Atf3* KO mice than WT mice 2 weeks post‐SCI. *n* = 6 or 7 in each group. Representative images of cresyl violet‐stained serial brain sections 3 days after permanent distal middle cerebral artery occlusion (pMCAO) (c) and their quantification (d) showing that *Atf3* KO mice had larger infarct volume than WT mice. Scale bar = 1 mm. *n* = 6 in each group. (e) *Atf3* KO mice had increased numbers of FJC^+^ degenerating neurons 3 days after stroke. *n* = 6 in each group. Data are presented as mean ± SEM and are analysed with unpaired two‐tailed *t*‐test, ^***^ *p* < .001.

To further investigate the functional role of ATF3 in the CNS, we examined the lesion size following SCI or pMCAO in both WT and *Atf3* KO mice. Two weeks after SCI, we evaluated the maximal lesion area at the injury epicentre using EC staining^30^ and observed that the ratio of ipsilateral lesion area to total contralateral uninjured area was significantly enlarged in *Atf3* KO compared to WT mice (Figure 7a,b). Additionally, we measured and compared infarct volumes in the brains collected 3 days after pMCAO, and we found that the *Atf3* KO mice had significantly larger infarct volumes than WT mice (Figure 7c,d).

### 3.10 *Atf3* KO mice exhibit more cortex neuronal degeneration after ischaemic stroke

We further demonstrated that *Atf3* KO mice had significantly more FJC^+^ degenerating neurons than WT mice 3 days after pMCAO in the peri‐infarct region (Figures 7e and S7). These data, in conjunction with those presented in Figure 7c,d, indicate that *Atf3* KO mice have more severe neural tissue damage than WT mice after SCI or ischaemic stroke. Taken together, our behavioural and histological results suggest that ATF3 has neuroprotective function after SCI and ischaemic stroke.

## 4 DISCUSSION

The current medical and surgical managements of CNS injuries, such as SCI and ischaemic stroke, relies on the prompt recognition of injuries to determine options for acute interventions. While neurological examination remains the primary tool for acute triage, it can be time‐consuming and dependent on patients’ cooperation. Although radiographic studies are often needed for injury diagnosis, they are not always available in acute clinical settings. Therefore, there is an urgent need to identify CNS injury biomarkers that clinicians can use to objectively assess patients’ initial injuries.

The use of biomarkers in clinical medicine has been successful in diagnosing tissue damage (cardiac enzyme levels), classifying tumours and assessing their severity (carcinoembryonic antigen and prostate‐specific antigen), and evaluating the risk of atherosclerotic cardiovascular disease risk and response to therapy (blood lipid profile after cholesterol‐lowering medication).^45^, ^46^, ^47^, ^48^ In this study, we demonstrate that ATF3, a member of CREB family transcription factor,^10^, ^11^ is specifically induced in spinal cord or cortex neurons shortly after traumatic SCI or ischaemic stroke, respectively. ATF3 protein levels are elevated in the blood in animal models of SCI and ischaemic stroke. Importantly, ATF3 is detectable and elevated in the serum of patients with SCI and ischaemic stroke. Furthermore, ATF3 exhibits a neuroprotective function after SCI or ischaemic stroke.

Biomarkers for CNS injuries have been extensively studied.^1^, ^2^, ^3^, ^4^, ^5^ In SCI, serum and cerebrospinal fluid, GFAP, NF, peripheral cytokine and chemokine levels,^6^, ^7^ and microRNA or RNA profiles in circulating leukocytes^3^ have been investigated as potential diagnostic and prognostic biomarkers. In TBI, blood levels of GFAP and UCH‐L1 have received approval from the Federal Drug Administration as biomarkers for intracranial lesion (haemorrhage, fracture, oedema) following mild TBI and concussion.^8^ However, when measured in combination, these biomarkers only help to identify the likelihood of imaging evidence of head injury rather than aiding in the clinical diagnosis of concussion or TBI.^8^ GFAP and S100b have also recently been investigated as point‐of‐care platform biomarkers for TBI patients.^9^ Similarly, GFAP, S100b and NSE, among others, have been investigated as the biomarkers for stroke.^5^

However, the existing biomarkers are proteins or RNAs that are either not expressed in neurons or expressed constitutively in all neurons. None of these biomarkers are specifically induced in CNS neurons following injury and have a low baseline expression level. Ideally, a biomarker with low baseline level but primarily induced in CNS neurons after injury would offer higher sensitivity and specificity in detecting the severity of neuronal injuries. In SCI and ischaemic stroke models, we found that ATF3 is not expressed in naive spinal cord or cortex. Instead, it is rapidly induced in CNS neurons specifically after CNS injuries. Importantly, we show that ATF3 protein is detectable and elevated in the blood shortly after SCI or ischaemic stroke (Figure 4). Our study holds considerable translational value because we demonstrate that it is feasible to measure ATF3 in human serum with a commercially available ELISA kit. Furthermore, serum ATF3 levels are elevated in clinical SCI and ischaemic stroke patients compared to control cohorts (Figure 5). It is noteworthy that serum ATF3 remains unchanged in peripheral trauma patients without spinal cord or brain injuries (Figure 5a), indicating that serum ATF3 serves as a biomarker for CNS injuries. It is unclear how ATF3 is released from the injured neurons into the bloodstream, although it is likely that ATF3 is released after the ATF3‐expressing neurons die.

Injuries to spinal cord and brain can be devastating, often resulting in permanent neurologic dysfunction.^49^, ^50^, ^51^, ^52^ One of the primary pathologies associated with CNS injuries is neuronal damage and death,^53^, ^54^, ^55^, ^56^, ^57^ which strongly correlates with injury severity and functional outcome. However, identifying cellular markers specifically for neuronal injury has proven challenging. For example, FJC^43^, ^58^, ^59^, ^60^ has been widely used to label degenerating neurons, but it is unclear whether FJC can effectively label injured neurons that have not yet degenerated. Indeed, FJC has never been reported to label injured but non‐degenerating sensory neurons after peripheral nerve injury. Caspases^53^, ^61^ and terminal deoxynucleotidyl transferase dUTP nick end labelling^62^ stains are also commonly used to label the neurons undergoing programmed cell death. However, these markers lack neuron specificity and are ineffective at detecting injured neurons that are not undergoing cell death. Unlike the degenerated or dying neurons labelled by current markers, early‐stage injured neurons that have not yet degenerated, and thus cannot be identified by existing markers, are more likely to recover from the injury. Therefore, they should be the primary cellular targets for therapeutic interventions.

The importance of cellular markers for injured neurons in understanding how neurons respond to injury has been clearly illustrated in a model of peripheral nerve injury, which has demonstrated robust post‐injury regenerative responses.^15^, ^63^ *Atf3* is induced in DRG sensory neurons after peripheral nerve injury, and it is widely recognised as a cellular marker of injured sensory neurons.^11^, ^12^, ^14^ This knowledge has remarkably facilitated the study on how sensory neurons respond to nerve injury. Using *Atf3* as a marker gene for injured DRG neurons, recent single cell RNA‐Seq analysis of DRG neurons has revealed the time course of gene expression profile change in the injured DRG neurons and the contribution of *Atf3* to the regulation of gene expression in DRG sensory neurons following peripheral nerve injury.^64^

Our current study demonstrates that ATF3 can also serve as a cellular marker for injured CNS neurons. ATF3 is exclusively induced in spinal cord neurons 1 day post‐SCI and in cortex neurons 1 day after ischaemic stroke. Interestingly, after a stroke, all FJC^+^ neurons were also ATF3^+^, but there were many ATF3^+^ neurons were FJC^−^. This suggests that ATF3 is likely more sensitive than FJC in detecting injured neurons. Furthermore, since FJC staining cannot even label degenerated spinal neurons after SCI,^44^ ATF3 has the advantage to be a specific marker for neuronal injury in SCI. Further research on these ATF3^+^ neurons is likely to enhance our understanding of how injured CNS neurons respond after injury. This research may also aid in the development of appropriate early interventions to rescue acutely injured CNS neurons.

As a member of CREB family transcriptional factors, ATF3 exhibits neuroprotective function in the peripheral nervous system. For instance, studies have demonstrated that *Atf3* overexpression promotes neurite outgrowth in cultured DRG neurons following axotomy^16^ and enhances axonal regeneration after peripheral nerve injury.^15^ In the zebrafish spinal cord transection model, knockdown of *Atf3* expression using anti‐sense *Atf3* morpholino resulted in reduced swimming distance and less axonal regrowth compared to control.^65^ In mouse TBI, global *Atf3* KO mice exhibit more prominent cerebral haemorrhage.^66^ Lentiviral overexpression of *Atf3* in cultured murine neurons also leads to a reduction in glutamate neurotoxicity.^67^ Furthermore, in a transgenic mouse model of amyotrophic lateral sclerosis, increased *Atf3* gene expression in motor neurons extended the survival of injured motor neurons.^68^

In this study, we observed a significantly larger injury area and more severe neurological deficits in *Atf3* KO mice compared to WT controls after SCI. Consistent with previous report,^69^ we also confirmed that *Atf3* KO mice experienced more extensive neurological damage following ischaemic stroke when compared to WT mice. Furthermore, there was a notable increase in the number of FJC^+^ degenerated neurons in *Atf3* KO mice compared to WT mice after ischaemic stroke. Further studies are needed to investigate the mechanism underlying ATF3's neuroprotective role in SCI and ischaemic stroke.

One limitation of our study is that we used the *Atf3* global KO mice, and thus we cannot rule out the contribution of ATF3 from the non‐neuronal cells such as microglia in SCI and ischaemic stroke. Another limitation is that we measured serum ATF3 in a relatively small number of SCI and ischaemic stroke patients. As a result, this study is only powered to demonstrate the elevation of serum ATF3 levels in moderate to severe SCI and ischaemic stroke patients. Nevertheless, our study successfully demonstrates the concept and the feasibility of measuring ATF3 level in patients’ serum samples as a potential biomarker. Further large‐scale clinical studies are warranted to validate the utility of serum ATF3 levels as a biomarker for SCI and ischaemic stroke. Furthermore, it would be intriguing to explore whether human serum ATF3 could also serve as a biomarker for other CNS injuries, such as TBI or haemorrhagic stroke, in future investigations.

## AUTHOR CONTRIBUTIONS

*Conceptualisation*: Jonathan Z. Pan, Jacqueline C. Bresnahan, Mervyn Maze, Xiangning Jiang, Michael S. Beattie, Hua Su and Zhonghui Guan. *Methodology*: Jonathan Z. Pan, Zhanqiang Wang, Wei Sun, Wei Li, Peipei Pan, Yongtao Sun, Shoulin Chen, Amity Lin, Wulin Tan, Liangliang He, Lijun An, Rich Liang, Pamela Fung, Qifeng Li, Nikolaos Kyritsis, Xuan Duong Fernandez, Sara Moncivais, Esmeralda Mendoza, Yuwen Chang, Zhaoyang Xiao, Gongming Wang, Qihang Du, Xinhuan Niu, Neel S. Singhal, Hua Su and Zhonghui Guan. *Investigation*: Jonathan Z. Pan, Zhanqiang Wang, Wei Sun, Shoulin Chen, Wei Li, Wulin Tan, Peipei Pan, Neel S. Singhal, Hua Su and Zhonghui Guan. *Visualisation*: Jonathan Z. Pan, Hua Su, Michael S. Beattie, Hua Su and Zhonghui Guan. *Funding acquisition*: Jonathan Z. Pan, Michael S. Beattie and Zhonghui Guan. *Project administration*: Jonathan Z. Pan, Michael S. Beattie, Hua Su and Zhonghui Guan. *Supervision*: Jonathan Z. Pan, Michael S. Beattie, Hua Su and Zhonghui Guan. *Writing—original draft*: Jonathan Z. Pan, Zhanqiang Wang, Peipei Pan, Michael S. Beattie, Hua Su and Zhonghui Guan. *Writing—review and editing*: all authors.

## CONFLICT OF INTEREST STATEMENT

The authors declare that they are applying for a patent based on this study.

## ETHICS STATEMENT

The animal studies were approved by the local institutional animal care and use program. The clinical procedures for the SCI study were conducted with the approval of the Human Subjects Review Boards at the University of California San Francisco, and the U.S. Department of Defense Human Research Protection Office (IRB number: 15‐16115). The clinical procedures for the stroke study were conducted with the approval of the Human Subjects Institutional Review Boards at the Zuckerberg San Francisco General Hospital and the University of California San Francisco (IRB number: 19‐27658).

## Supporting information

Supporting Information

Supporting Information

Supporting Information

## Data Availability

For additional information and requests for resource and reagents, please contact the lead investigator, Dr. Zhonghui Guan (zhonghui.guan@ucsf.edu). This study did not generate new unique reagents. RNA‐Seq data can be accessed at Gene Expression Omnibus (GSE180767) with the reviewer token ‘ebmzswwstvuhngt’.
