# Supplementary material for: ATF3 is a neuron‐specific biomarker for spinal cord injury and ischaemic stroke
Source: Clin Transl Med. 2024 Apr 22;14(4):e1650. doi: 10.1002/ctm2.1650 (PMC11035380; doi:10.1002/ctm2.1650)
Supplement: Supplementary file 1 — Supporting Information [file CTM2-14-e1650-s001.docx]

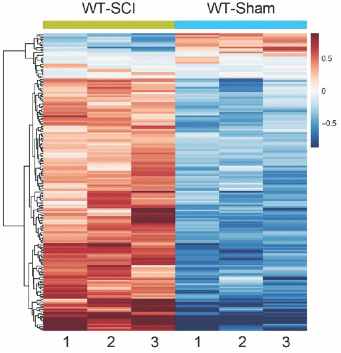


**Supplementary Figure 1. *RNA-Seq* of mouse spinal cord after spinal cord injury.**

Heat map of mouse spinal cord RNA-Seq results before (sham) and 4 hours after SCI showing 177 differentially expressed genes (DEG) with more than 1.5-fold changes (160 upregulated and 17 downregulated).


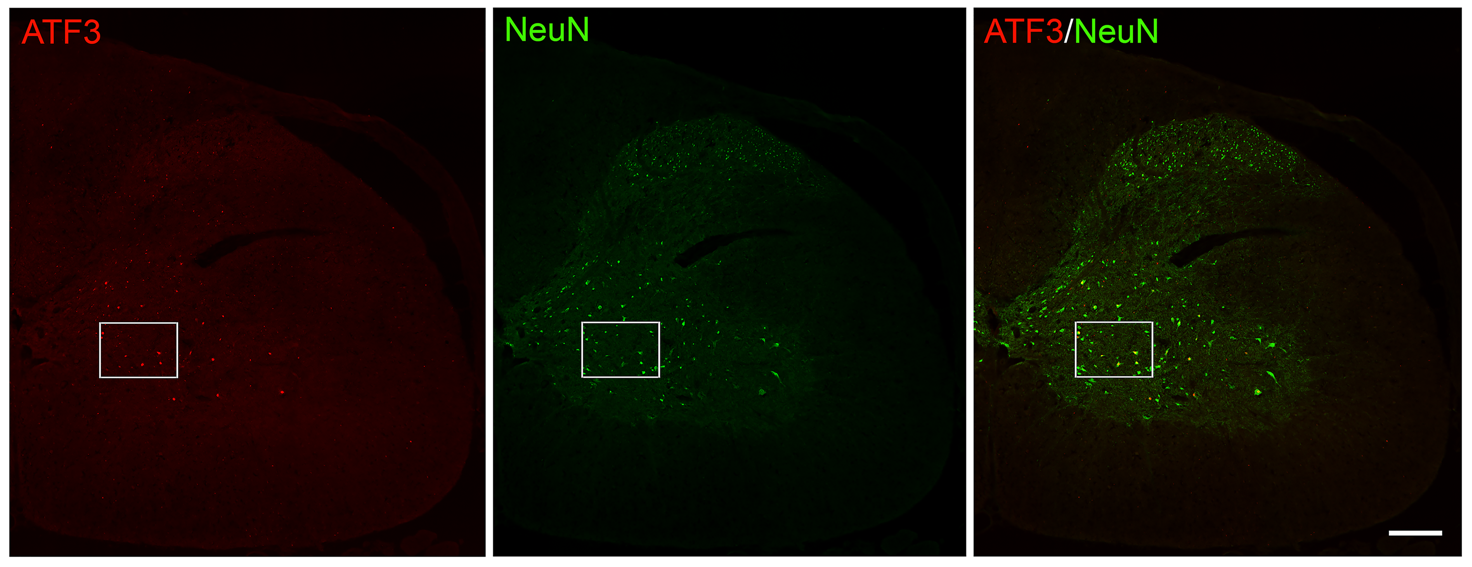
a

b

**
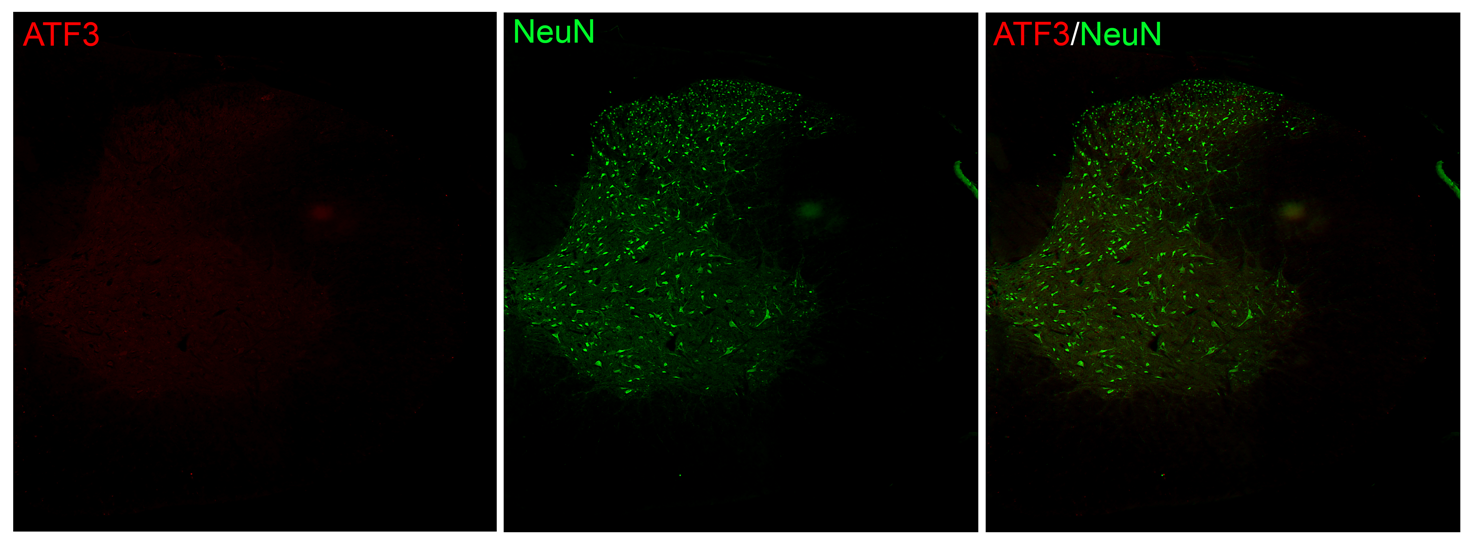
**

**Supplementary Figure 2. ATF3 is only expressed in neurons 1 day after SCI.**

a: Representative immunohistochemical staining of NeuN and ATF3 in injured hemi-cord 1day post injury (4x). ATF3 expression is detected in the NeuN^+^ neurons in the gray matter of injured hemi-cord. Higher power images in the marked area are shown in Figure 2. b: Uninjured spinal cord control, NeuN+ neurons have no expression of ATF3. Scale bar = 200 μm.


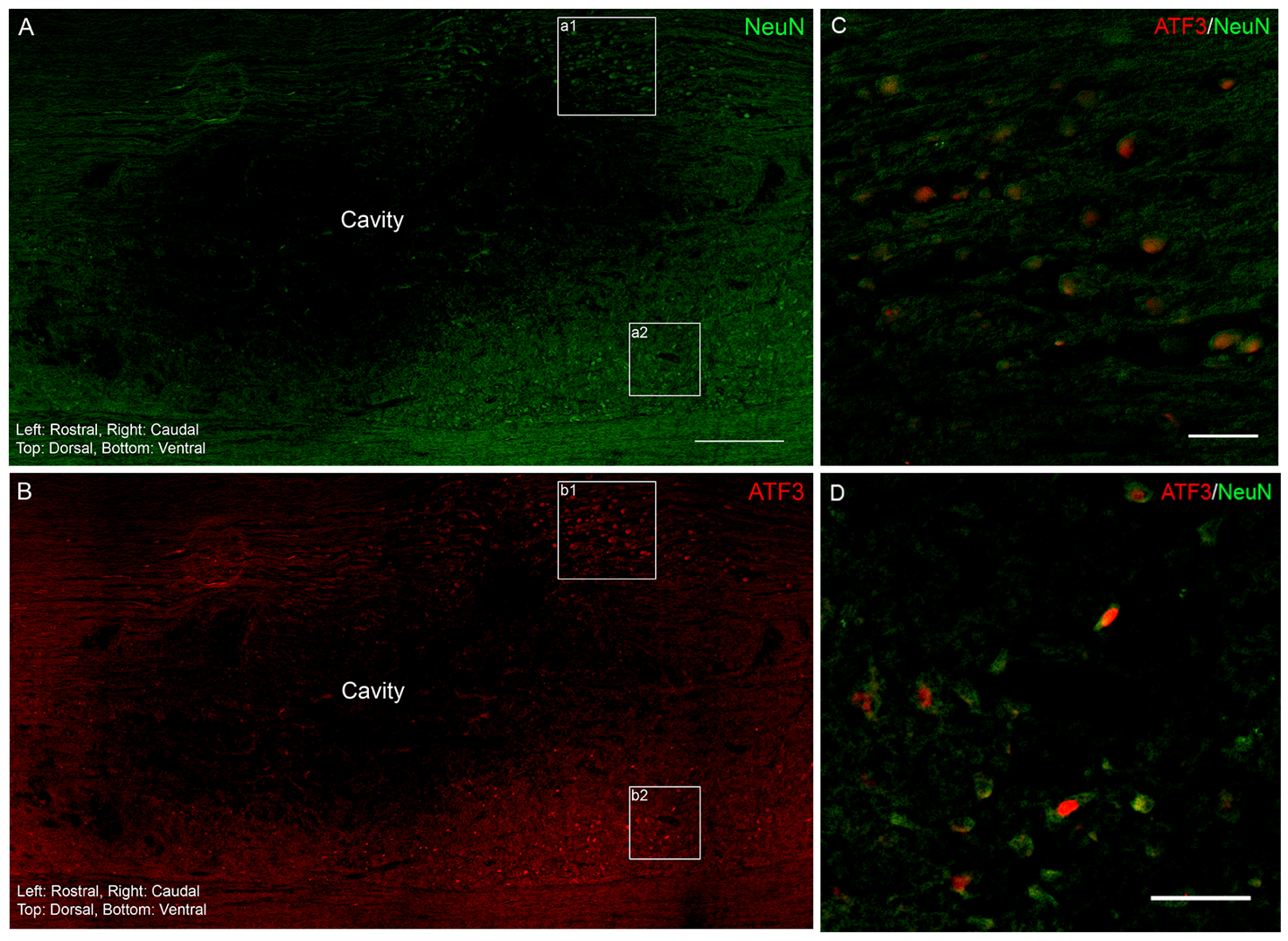


**Supplementary Figure 3. ATF3 induction in the area surrounding the center of the injury 1 day after SCI.**

Representative immunohistochemical staining of NeuN and ATF3 in longitudinal injured hemi-cord 1 day post injury (A and B). ATF3 expression is detected in NeuN+ neurons in the area surrounding the center of the damage in the injured hemi-cord, C (a1+b1) and D (a2+b2). The centered dark region is the cavity of the injury. Scale bar = 300 μm for A and B, 50 μm for C and D.


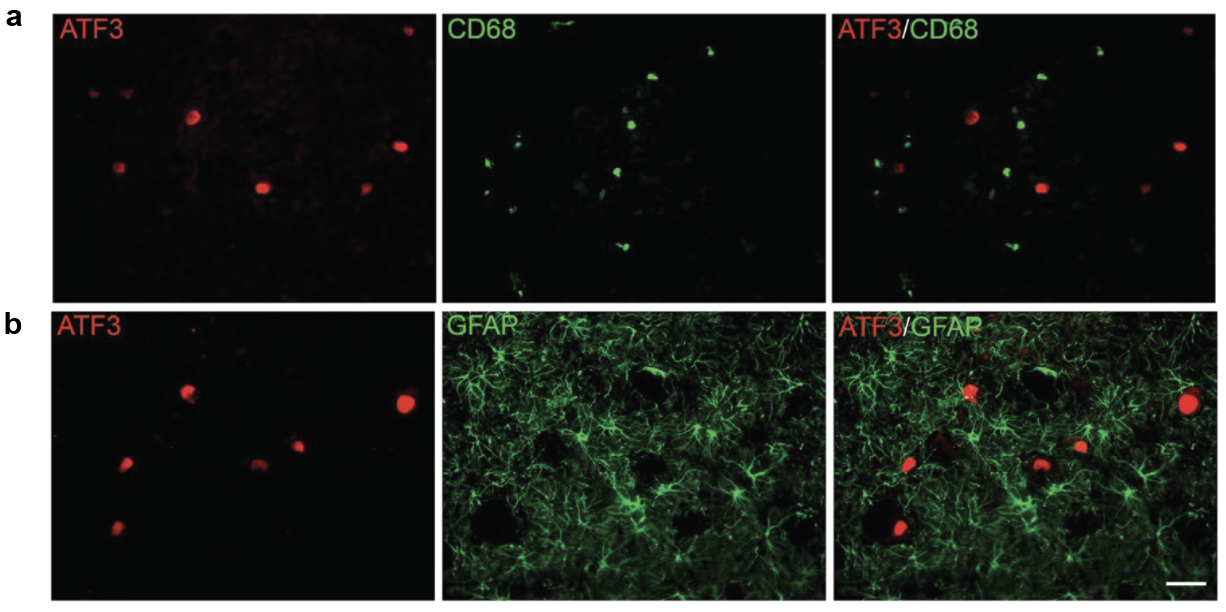


**Supplementary Figure 4. ATF3 is not expressed in spinal cord microglia or astrocyte 1 day after SCI.**

Representative immunohistochemical staining of ATF3 and CD68 or GFAP in injured hemi-cord 1 day after SCI. ATF3 is not expressed in microglia/macrophages (CD68+ cells, **a**) or astrocytes (GFAP+ cells, **b**). Scale bar = 40 μm. The images are the magnification of the squared areas in Supplementary Figure 2.


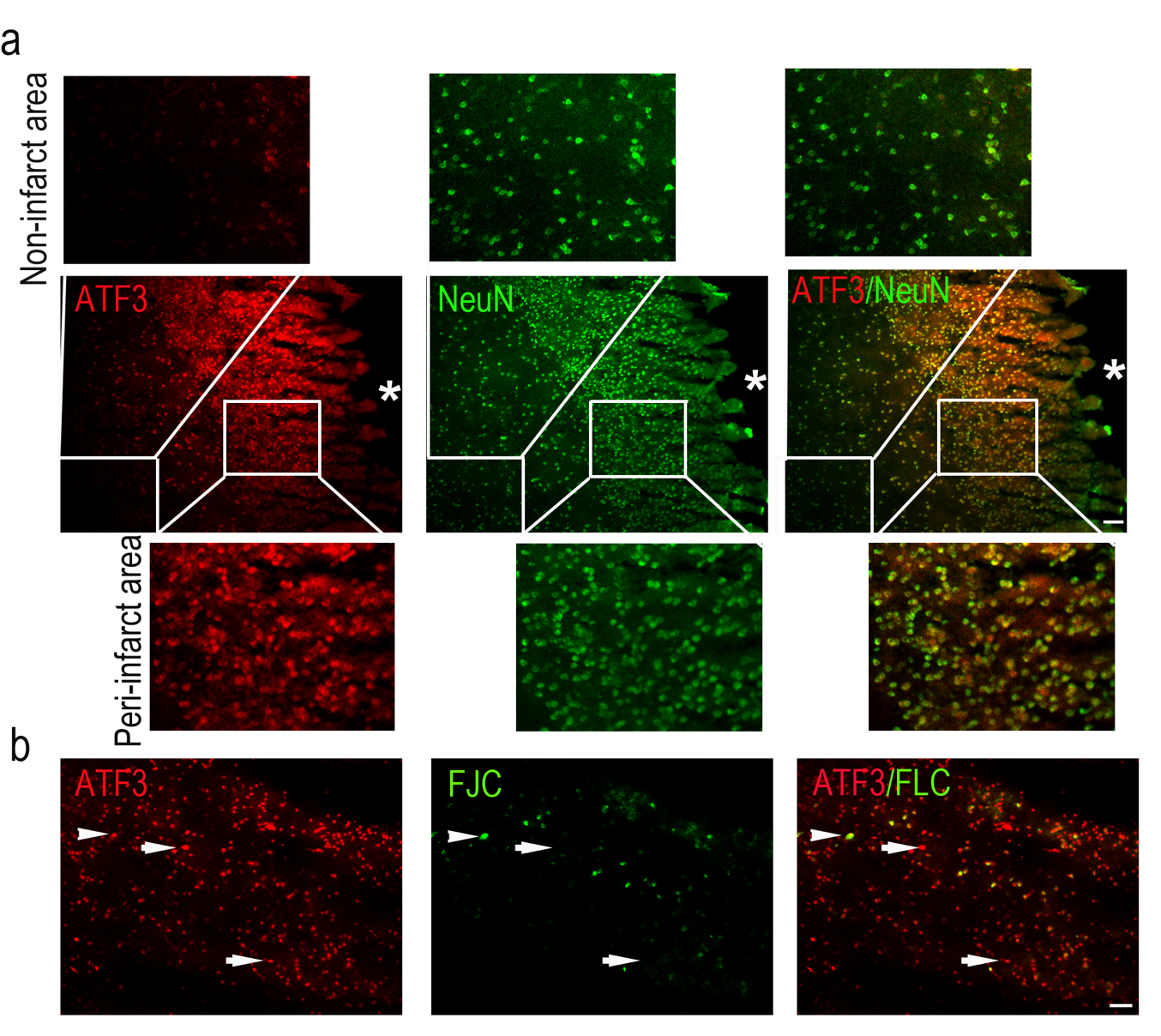


**Supplementary Figure 5. ATF3 is induced in the neurons in the peri-infarct area 1 day after ischemic stroke.**

**a.** Representative immunohistochemical staining of NeuN and ATF3 in the peri-infarct and adjacent areas of a mouse brain with ischemic injury 1 day after pMCAO. All ATF3^+^ cells are NeuN^+^. *: indicted the infarct side. The peri-infarct area (bottom) and non-infarct area (top) are enlarged to show the details. Scale bar = 100 μm.

**b.** Representative immunohistochemical staining of ATF3 and Fluoro Jade C (FJC, a known marker for degenerating neurons) in the peri-infarct ischemia region 1 days after pMCAO in mice. All FJC^+^ cells are ATF3^+^ (arrowheads), but some ATF3^+^ cells are FJC^-^ (arrows). Scale bar = 50 μm.

pMCAO: permanent distal middle cerebral artery occlusion.


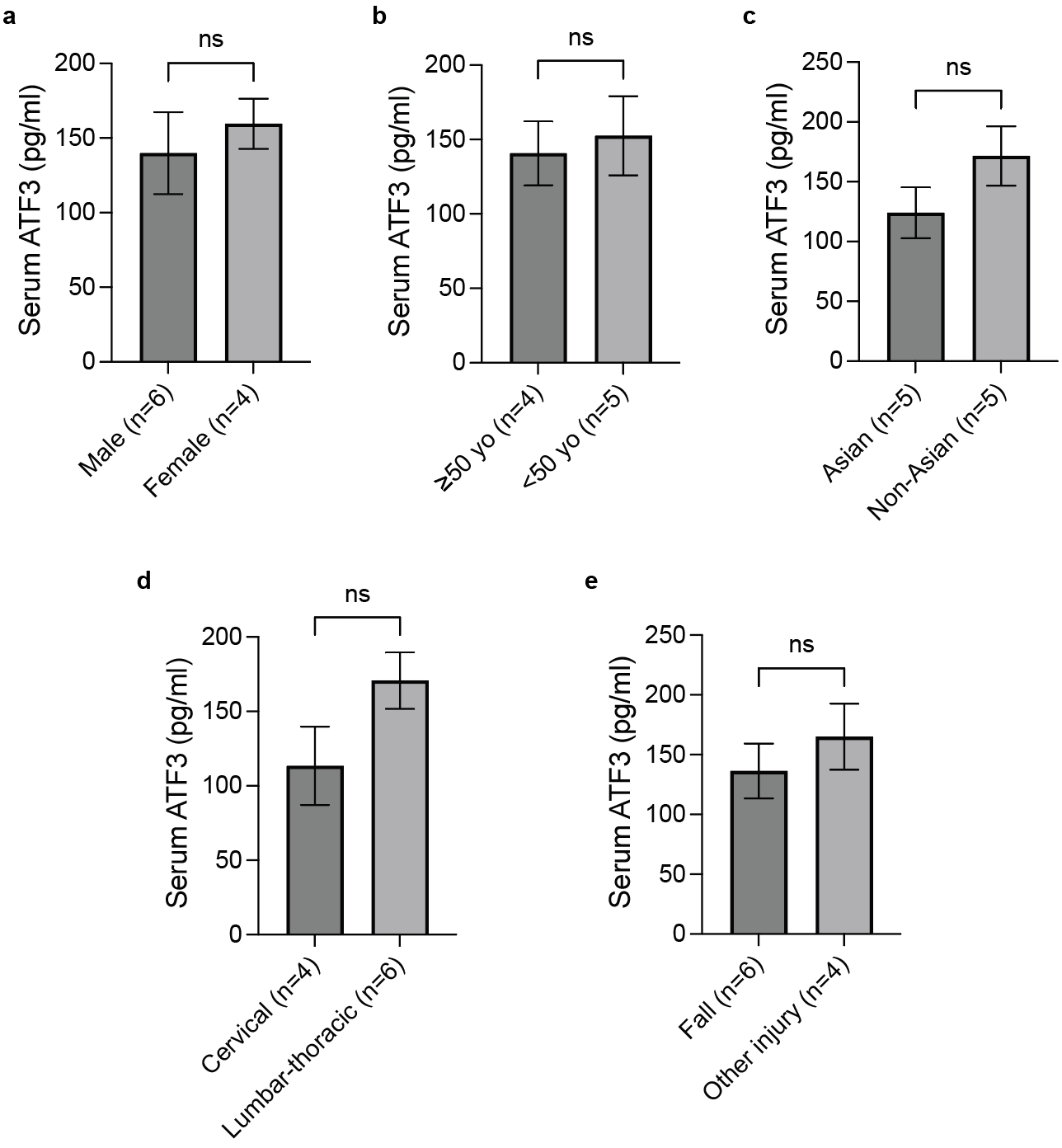
**Supplementary Figure 6. Serum ATF3 level in SCI patients 24 hours after injury.**

In AIS A (the most severe) SCI patients, there were no statistically significant differences in serum ATF3 levels among sexes (**a**), ages (**b**), races (**c**), injury levels (**d**), or injury categories (**e**).

Data are presented as mean ± S.E.M. and were analyzed with unpaired two-tailed t-test, “ns” as no statistical significance.

**
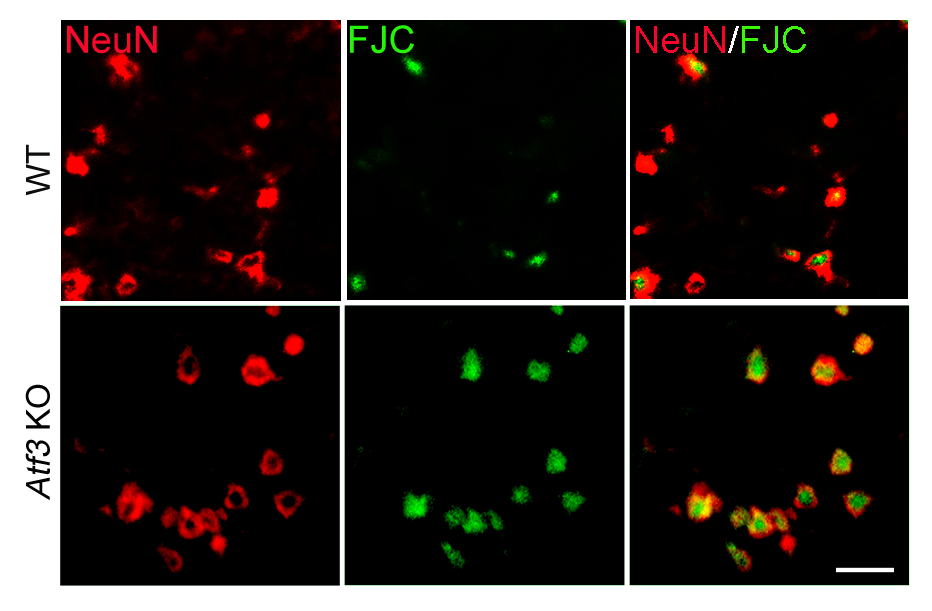
**

**Supplementary Figure 7. *Atf3* knockout mice have more degenerated cortex neurons after ischemic stroke.**

Representative immunohistochemical images of NeuN and FJC peri-infarct ischemia region 3 days after pMCAO. Scale bar = 50μm.
