# Supplementary material for: ATF3 is a neuron‐specific biomarker for spinal cord injury and ischaemic stroke
Source: Clin Transl Med. 2024 Apr 22;14(4):e1650. doi: 10.1002/ctm2.1650 (PMC11035380; doi:10.1002/ctm2.1650)
Supplement: Supplementary file 2 — Supporting Information [file CTM2-14-e1650-s002.docx]

**Supplementary Table 1. Demographic data of spinal cord injury patients**

|  | **AIS A** | **AIS B** | **AIS C** | **AIS D** | **Trauma control** | **Healthy control** |
| --- | --- | --- | --- | --- | --- | --- |
| n | 10 | 5 | 5 | 10 | 7 | 7 |
| Age, Mean (SD) | 47.5 (4.3) | 50.2 (10.2) | 65.2 (9.2) | 56.2 (5.6) | 42.9 (3.9) |  |
| Sex, No. (%) |  |  |  |  |  |  |
| Male | 6 (60) | 5 (100) | 3 (60) | 7 (70) | 3 (43) | 6 (86) |
| Female | 4 (40) | 0 (0) | 2 (40) | 3 (30) | 4 (57) | 1 (14) |
| Race, No. (%) |  |  |  |  |  |  |
| Asian | 5 (50) | 0 (0) | 0 (0) | 3 (30) | 1 (14) | 3 (43) |
| Black | 2 (20) | 2 (40) | 0 (0) | 0 (0) | 0 (0) | 1 (14) |
| White | 2 (20) | 1 (20) | 3 (60) | 4 (40) | 4 (57) | 3 (43) |
| Hispanic | 1 (10) | 1 (20) | 2 (40) | 1 (10) | 1 (14) | 0 (0) |
| Native Hawaiian | 0 (0) | 0 (0) | 0 (0) | 1 (10) | 0 (0) | 0 (0) |
| Other | 0 (0) | 1 (20) | 0 (0) | 1 (10) | 1 (14) | 0 (0) |
| Level of injury, No. (%) |  |  |  |  |  |  |
| Cervical | 4 (40) | 4 (80) | 5 (100) | 7 (100) | NA | NA |
| Thoracic | 4 (40) | 1 (20) | 0 (0) | 0 (0) | NA | NA |
| Lumbar | 2 (20) | 0 (0) | 0 (0) | 0 (0) | NA | NA |
| Injury Mechanism, No. (%) |  |  |  |  |  |  |
| Fall | 6 (60) | 1 (20) | 5 (100) | 8 (80) | NA | NA |
| Gun shot | 2 (20) | 0 (0) | 0 (0) | 0 (0) | NA | NA |
| Hit by tree | 1 (10) | 0 (0) | 0 (0) | 0 (0) | NA | NA |
| MCC | 0 (0) | 2 (40) | 0 (0) | 0 (0) | NA | NA |
| MVA | 0 (0) | 0 (0) | 0 (0) | 1 (10) | NA | NA |
| MVC | 0 (0) | 1 (20) | 0 (0) | 1 (10) | NA | NA |
| PVA | 1 (10) | 1 (20) | 0 (0) | 0 (0) | NA | NA |

MCC: Motorcycle Crash; MVA: Motor Vehicle Accident; MVC: Motor Vehicle Collison; PVA: Pedestrian Vehicle Accident
