# Supplementary material for: ATF3 is a neuron‐specific biomarker for spinal cord injury and ischaemic stroke
Source: Clin Transl Med. 2024 Apr 22;14(4):e1650. doi: 10.1002/ctm2.1650 (PMC11035380; doi:10.1002/ctm2.1650)
Supplement: Supplementary file 3 — Supporting Information [file CTM2-14-e1650-s003.docx]

**Supplementary Table 2. Demographic data of 21 ischemic stroke patients**

| Age, Mean (SD) | 76.7 (12.7) |
| --- | --- |
| Sex, No. (%) |  |
| Male | 9 (42.9) |
| Female | 12 (57.1) |
| Race, No. (%) |  |
| Asian | 12 (57.1) |
| Black | 1 (4.8) |
| White | 2 (9.5) |
| Hispanic | 5 (23.8) |
| Native Hawaiian | 1 (4.8) |
| Stroke risk factor, No. (%) |  |
| Hypertension | 14 (66.7) |
| Atrial fibrillation | 8 (38.1) |
| Diabetes | 1 (4.8) |
| Smoking | 4 (19.0) |
| Hyperlipidemia | 15 (71.4) |
| Stroke type, No. (%) |  |
| Large-artery atherosclerosis | 3 (14.3) |
| Cardioembolism | 5 (23.8) |
| Small-vessel occlusion | 3 (14.3) |
| Undetermined etiology | 10 (47.6) |
| Thrombectomy performed | 3 (14.3) |
| tPA administered | 5 (23.8) |

tPA: tissue plasminogen activator
